# Cartridge-Based Thromboelastography Can Be Used to Monitor and Quantify the Activity of Unfractionated and Low-Molecular-Weight Heparins

**DOI:** 10.1055/s-0039-1696658

**Published:** 2019-09-12

**Authors:** João D. Dias, Carlos G. Lopez-Espina, Mauro Panigada, Heidi J. Dalton, Jan Hartmann, Hardean E. Achneck

**Affiliations:** 1 Haemonetics Corporation, Signy, Switzerland; 2 Haemonetics Corporation, Rosemont, Illinois, United States; 3 Fondazione IRCCS Ca' Granda - Ospedale Maggiore Policlinico, Milan, Italy; 4 Department of Pediatrics, Inova Health and Vascular Institute, Falls Church, Virginia, United States; 5 Haemonetics Corporation, Braintree, Massachusetts, United States

**Keywords:** thromboelastography, point-of-care diagnostic system, low-molecular-weight heparin, heparin

## Abstract

Thromboelastography is increasingly utilized in the management of bleeding and thrombotic complications where heparin management remains a cornerstone. This study assessed the feasibility of the cartridge-based TEG ^®^ 6s system (Haemonetics Corp., Braintree, Massachusetts, United States) to monitor and quantify the effect of unfractionated and low-molecular-weight heparin (UFH and LMWH). Blood samples from healthy donors were spiked with UFH ( *n* = 23; 0–1.0 IU/mL) or LMWH (enoxaparin; *n* = 22; 0–1.5 IU/mL). Functional fibrinogen maximum amplitude (CFF.MA), RapidTEG activated clotting time (CRT.ACT), and kaolin and kaolin with heparinase reaction time (CK.R and CKH.R) were evaluated for their correlation with heparin concentrations, as well as the combination parameters ΔCK.R − CKH.R, ratio CK.R/CKH.R, and ratio CKH.R/CK.R. Nonlinear mixed-effect modelling was used to study the relationship between concentrations and parameters, and Bayesian classification modelling for the prediction of therapeutic ranges. CK.R and CRT.ACT strongly correlated with the activity of LMWH and UFH ( *p* < 0.001). Using combination parameters, heparin activity could be accurately quantified in the range of 0.05 to 0.8 IU/mL for UFH and 0.1 to 1.5 IU/mL for LMWH. CRT.ACT was able to quantify heparin activity at higher concentrations but was only different from the reference range ( *p* < 0.05) at >0.5 IU/mL for UFH and >1.5 IU/mL for LMWH. Combination parameters classified blood samples into subtherapeutic, therapeutic, and supratherapeutic heparin ranges, with an accuracy of >90% for UFH, and >78% for LMWH. This study suggests that TEG 6s can effectively monitor and quantify heparin activity for LMWH and UFH. Additionally, combination parameters can be used to classify blood samples into therapeutic ranges based on heparin activity.

## Introduction

Heparin is a mainstay of antithrombotic therapy used primarily for systemic anticoagulation and for the treatment or prevention of conditions such as thromboembolism. 1 2 The anticoagulant effect of heparin is a result of binding to antithrombin, resulting in a conformational change which increases antithrombin activity and therefore inhibition of coagulation factors, including factor (F) Xa and IIa (thrombin). Unfractionated heparin (UFH) is composed of sulfated glycosaminoglycans with molecular weights varying from approximately 3 to 30 kDa. The heterogeneity of the heparin molecules present in UFH allows it to promote the inhibition of more proteases than any single heparin molecule; however, it also results in variable bioactivity and patient response. 1 3 4 An alternative is low-molecular-weight heparins (LMWHs), which are produced by fractionating heparin through chemical or enzymatic cleavage. 1 4 This process results in a more homogenous preparation consisting of fragments with a lower molecular weight and more predictable action than UFH.

To avoid under- or overdosing with heparin, it is vital to monitor the degree of anticoagulation carefully. While laboratory monitoring of UFH is widely recommended to maintain the target therapeutic dose, monitoring of LMWH is only recommended in patients with unpredictable pharmacokinetics, such as those who are obese or in renal failure. 1 4 5 6 The principal method used to monitor the activity of UFH is activated partial thromboplastin time (aPTT). 7 In contrast, the anti-Xa activity assay is of particular value for monitoring LMWHs, as they predominantly inhibit FXa, 1 7 and may also be superior to aPTT for monitoring UFH. 8 9 10 11 However, the anti-Xa activity assay is less available and less familiar to clinicians, more expensive than aPTT, and requires samples to be processed within 1 hour to avoid heparin neutralization from platelet factor 4. 11 12 In addition, as the test is usually conducted in the laboratory, results can take longer to obtain than those available at point of care.

TEG ^®^ (Haemonetics Corp., Braintree, Massachusetts, United States) is a viscoelastic point-of-care diagnostic system used for monitoring clot formation and hemostasis. 13 14 15 16 17 TEG can be used to monitor the antithrombotic effects of UFH and LMWHs, and has been shown to be more sensitive to the level of anticoagulation than conventional laboratory tests. 18 19 20 The new TEG 6s is a fully automated instrument that employs an all-in-one four-channel cartridge, enabling a rapid assay preparation time of less than 1 minute. 16 21 22 23 Compared with the TEG 5000, the TEG 6s offers simplified assay preparation and a reduced required blood volume. 15 22 The channels in the TEG 6s cartridge perform different assays, including kaolin with heparinase (CKH), which is the only assay that contains heparinase to reverse heparin and reveal the underlying coagulation profile. 15 24 Studies have previously demonstrated the effectiveness of TEG in the treatment and diagnosis of heparin-induced coagulopathy. 14 19 Additionally, extracorporeal membrane oxygenation (ECMO) guidelines note the usefulness of TEG, as it can provide information relevant to multiple stages of coagulation. 25 26 However, there are currently only a limited number of studies that have investigated TEG parameter combinations to monitor heparin, with no consensus reached. 25 27 28 29 The aim of this study was to assess whether the TEG 6s system can be used to monitor and quantify concentrations of UFH and LMWH in blood, to evaluate the use of combination TEG 6s parameters, and to confirm whether these parameters can effectively classify blood samples at subtherapeutic, therapeutic, and supratherapeutic levels of heparin.

## Methods

### Study Design

This study was conducted using blood samples from healthy donors (LMWH group, *n* = 22; UFH group, *n* = 23), following guidelines in CLSI C28-A3c, Good Laboratory Practices (GLP), and methods previously referenced in TEG user manuals and validation guides. 30 Samples were extracted using antecubital venipuncture and a 21-gauge butterfly needle. Individuals with diabetic and metabolic syndromes, coagulation or thrombotic disorders, and those who had taken platelet-altering medications such as aspirin, ibuprofen, naproxen, and cold medications within the previous 2 days were excluded. Written informed consent was obtained from each participant before blood withdrawal. A total of 40 mL blood per donor was drawn using eight 4.5 mL Becton Dickinson (BD) Vacutainer Citrate Tubes with 3.2% buffered sodium citrate solution. Blood samples of 3.5 mL were prepared by spiking whole blood ex vivo with 12 concentrations of heparin ranging from either 0 to 1 IU/mL for UFH or 0 to 1.5 IU/mL for LMWH (enoxaparin; LOVENOX). The conversion rate between mg and IU mg/mL for enoxaparin was 100 IU/mL. Samples were then incubated at room temperature for 30 minutes prior to running in the TEG 6s to ensure that the added heparin had fully affected the blood sample. The samples were analyzed using the conventional coagulation tests aPTT and anti-Xa; results of the aPTT, anti-Xa, and TEG 6s tests are given in Supplementary Table S1 . As some samples fell below the limit of detection, not all samples were processed with anti-Xa.

### TEG 6s Analysis

Test samples were analyzed using the TEG 6s hemostasis analyzer (Haemonetics Corporation, Braintree, Massachusetts, United States); technical details of the TEG system have been described elsewhere. 15 Each analyzer was verified to be within its calibration period prior to running samples for this study, and cartridges were verified to be within their expiration dates. The TEG 6s employs a citrated multichannel cartridge containing four channels that perform different assays: (1) functional fibrinogen (CFF), (2) RapidTEG (CRT), (3) kaolin (CK), and (4) CKH. 15 16 22 The parameters analyzed for these assays included the reaction time (R), maximum amplitude (MA), and activated clotting time (ACT).

### Statistical Methods

Statistical methodology included generalized linear and nonlinear mixed-effect modelling to study the relationships between the UFH and LMWH concentrations and the individual TEG parameters. A Bayesian multinomial multilevel modelling framework was used to predict the therapeutic ranges of heparin, using combinations of the reaction times for CK and CKH (CK.R and CKH.R) as either the difference between or a ratio of these parameters (ΔCK.R − CKH.R, ratio CK.R/CKH.R, and ratio CKH.R/CK.R). Traditional coagulation test data (aPTT, anti-Xa) were also analyzed in a similar way and used to confirm that the samples had relevant levels of heparin.

To assess the effect of UFH and LMWH concentrations on each TEG 6s assay parameter, parameters were modeled with either mixed-effects (three- or four-parameter) logistic models or linear mixed-effects models. The following TEG 6s parameters were assessed: reaction time for CK and CKH (CK.R, CKH.R, and ΔCK.R − CKH.R); MA for CK, CKH, CRT, and CFF (CK.MA, CKH.MA, CRT.MA, and CFF.MA), and ACT for CRT (CRT.ACT). Each heparin type was analyzed with a separate model due to the dependency of LMWHs on the allosteric mechanism of heparin, which made concentration comparison incompatible. The heparin concentration was set as the fixed effect and the donor was set as the random effect to account for the occurrence of inter-donor variation. Any parameter results that were not produced were marked as “censored” in the statistical analyses and plots. Results for missing R-times, or R-times >60 minutes, were imputed as 60 minutes as this is the maximum time allowed by the instrument to quantify the parameter; missing MAs were imputed as zero. In the case of the anti-Xa assays, for baseline samples with no heparin, the results were imputed as zero. The quantification range for heparin for each parameter was determined. The minimum value was estimated as the first dose where the response was statistically significant from the dose 0 response; statistical significance was judged by testing the slope of the linear approximation between the two doses under consideration versus horizontal line, using the *p* -value of 0.05 as a critical value. The maximum value (saturation point) was estimated as the last dose where the response was statistically different from the last observed dose in the model; statistical significance was judged as for the minimum value. The reference range cutoff dose was estimated as the first dose that provided a statistically different response from the upper/lower fence of the reference range; the estimation was conducted by intersecting the reference range with the confidence interval of the fitted model results. Reference ranges for each parameter are detailed in Table 1 .

**Table 1:** Reference ranges for TEG 6s assay parameters

Assay	Parameter	Lower limit	Upper limit
CK	R	4.6	9.1
	MA	52	69
CKH	R	4.3	8.3
	MA	52	69
CRT	ACT	82	152
	MA	52	70
CFF	MA	15	32

Abbreviations: ACT, activated clotting time; CFF, citrated functional fibrinogen; CK, kaolin; CKH, kaolin with heparinase; CRT, RapidTEG; MA, maximum amplitude; R, reaction time.

To identify TEG combination metrics based on CK.R and CKH.R in relation to sample heparin levels, levels were based on anti-Xa assay results identified for UFH (subtherapeutic, <0.3 IU/mL; therapeutic, 0.3–0.7 IU/mL; supratherapeutic, >0.7 IU/mL) and LMWH (subtherapeutic, <0.5 IU/mL; therapeutic, 0.5–1.2 IU/mL; supratherapeutic, >1.2 IU/mL). 31 32 33 34 For the TEG combination metric analysis, when low concentration values were missing they were imputed as anti-Xa <0.2 IU/mL and classified as subtherapeutic. When high concentration values were missing, they were imputed as anti-Xa >2 IU/mL and classified as supratherapeutic. Three TEG R-time combination parameters were considered: (1) ΔCK.R − CKH.R; (2) ratio CK.R/CKH.R; and (3) ratio CKH.R/CK.R. The relationship between dependent variable classes (heparin samples) and the covariates (TEG R-time combination parameters) was estimated using a multilevel multinomial logistic regression model. The estimation of model parameters was performed using a Bayesian Markov chain Monte Carlo approach. The categorical prediction power of the new metrics was assessed for accuracy, level-specific specificity, sensitivity, and positive and negative predictive values (PPV and NPV). Once the model was established, cutoffs were obtained for the covariate.

## Results

Donor blood samples were spiked with either UFH ( *n* = 23) or LMWH ( *n* = 22). In the UFH group, three donors had missing anti-Xa assay data and a single donor was tested twice, albeit on different days. Anti-Xa assay data were missing for two donors in the LMWH group. The anticoagulant effect of heparin was confirmed by the anti-Xa assay, and increased levels of heparin resulted in higher values for anti-Xa with a maximum level of detection of 2 IU/mL.

### The Effect of UFH on TEG 6s Parameters

**Fig. 1:**
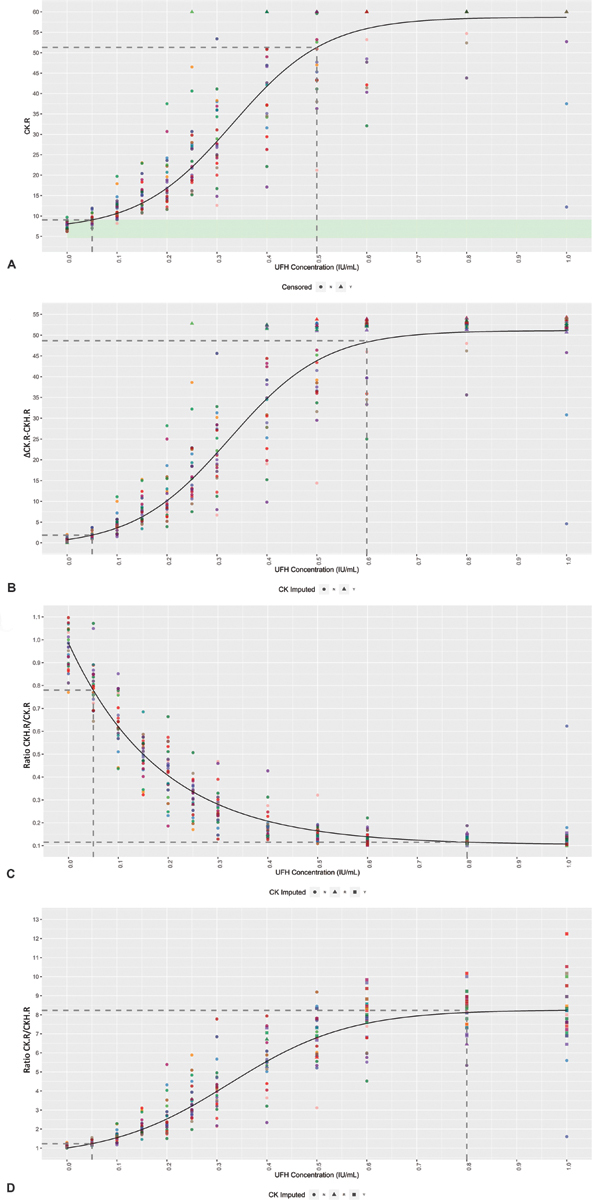
Concentration-dependent effects of UFH on the TEG 6s parameters. Mixed-effect four-parameter logistic model of ( **A** ) CK.R, ( **B** ) ΔCK.R − CKH.R, ( **C** ) ratio CKH.R/CK.R, and ( **D** ) ratio CK.R/CKH.R in relation to the UFH concentration. Individual donors are highlighted in different colors, with the model ( *black line* ) included. Reference range limits are shown in *light green* . The *dotted lines* represent the minimum and maximum values (first dose where the response was statistically significant from the dose 0 response or last dose where the response was statistically different from the last observed dose in the model, respectively). Values greater than 60 are beyond the limit of detection, and so are marked as censored. CK, kaolin; CKH, kaolin with heparinase; R, reaction time; UFH, unfractionated heparin.

Concentration-dependent effects of UFH were shown for the CK.R results ( Fig. 1A ; *p* < 0.001). Readings for the CK assay were within reference range limits for therapeutic levels of heparin, and higher UFH concentrations were associated with higher CK.R results as shown by a positive Spearman correlation of *ρ* = 0.929 ( *p* < 0.001). The first dose at which a significant difference from the reference range for CK.R was observed was 0.1 IU/mL. No statistically significant effects of UFH were shown on CKH.R assay results (slope *β* = 0.117, *p* = 0.3181). As for CK.R, higher concentrations of UFH tended to give higher results for the combination parameters ΔCK.R − CKH.R and ratio CK.R/CKH.R, as shown by positive Spearman correlations of *ρ* = 0.929 ( *p* < 0.001) and *ρ* = 0.901 ( *p* < 0.001), respectively ( Fig. 1B, D ). In contrast, higher concentrations of UFH resulted in lower results for ratio CKH.R/CK.R, with a negative Spearman correlation of *ρ* = − 0.901 ( *p* < 0.001; Fig. 1C ).

UFH activity could be accurately quantified in the range of 0.05 to 0.5 IU/mL for CK.R, 0.05 to 0.6 IU/mL for ΔCK.R − CKH.R, and 0.05 to 0.8 IU/mL for both ratio CKH.R/CK.R and ratio CK.R/CKH.R ( Table 2 ).

**Table 2:** Minimum and maximum quantification window for UFH and LMWH for the combination parameters and CRT.ACT

	UFH			LMWH		
	Minimum (IU/mL)	Reference range cutoff	Maximum (IU/mL)	Minimum (IU/mL)	Reference range cutoff	Maximum (IU/mL)
Ratio CKH.R/CK.R	0.05	No range	0.8	0.1	No range	1.5
Ratio CK.R/CKH.R	0.05	No range	0.8	0.1	No range	1.5
ΔCK.R − CKH.R	0.05	No range	0.6	0.1	No range	1.5
CRT.ACT	0.2	0.5	1	0.4	1.5	1.5

Abbreviations: ACT, activated clotting time; CK, kaolin; CKH, kaolin with heparinase; CRT, RapidTEG; LMWH, low-molecular-weight heparin; R, reaction time; UFH, unfractionated heparin.

In the therapeutic range of UFH versus anti-Xa (0.3–0.7 IU/mL), 91% of samples were accurately categorized by ratio CKH.R/CK.R, 90% by ratio CK.R/CKH.R, and 92% by ΔCK.R − CKH.R, with a PPV of 88% for ΔCK.R − CKH.R and ratio CK.R/CKH.R, and 89% for ratio CKH.R/CK.R ( Table 3 ), demonstrating that blood samples could be accurately classified according to clinically relevant levels of UFH. Samples not in this range were identified with a NPV of >95% for ratio CKH.R/CK.R, >96% for ratio CK.R/CKH.R, and >96% for ΔCK.R − CKH.R. Within the therapeutic window, samples were classified with a sensitivity of 72, 67, and 78%, and a specificity of 98, 98, and 97% for ratio CKH.R/CK.R, ratio CK.R/CKH.R, and ΔCK.R − CKH.R, respectively. Samples outside this window were classified with a sensitivity of >92, >93, and >94%, and a specificity of >92, >92, and >94% for ratio CKH.R/CK.R, ratio CK.R/CKH.R, and ΔCK.R − CKH.R, respectively.

**Table 3:** TEG combination parameters ratio CKH.R/CK.R, ratio CK.R/CKH.R, and ΔCK.R − CKH.R versus anti-Xa for UFH and LMWH

Heparin	Combination parameter		Cutoff		Subtherapeutic vs. anti-Xa				Therapeutic vs. anti-Xa				Supratherapeutic vs. anti-Xa			
		Accuracy (%)	Low	High	Sensitivity (%)	Specificity (%)	PPV (%)	NPV (%)	Sensitivity (%)	Specificity (%)	PPV (%)	NPV (%)	Sensitivity (%)	Specificity (%)	PPV (%)	NPV (%)
UFH	Ratio CKH.R/CK.R	91	0.34	0.20	92	95	92	95	72	98	89	93	98	92	90	99
	Ratio CK.R/CKH.R	90	3.67	5.27	93	92	88	96	67	98	88	92	98	94	92	99
	ΔCK.R − CKH.R	92	19	33	94	94	91	96	78	97	88	95	97	97	96	98
LMWH	Ratio CKH.R/CK.R	80	0.70	0.43	88	95	86	96	68	86	71	85	85	88	85	89
	Ratio CK.R/CKH.R	78	1.45	2.59	93	90	76	97	65	85	68	84	80	92	89	86
	ΔCK.R − CKH.R	79	3.45	12.99	91	92	78	97	65	85	68	84	82	91	87	87

Abbreviations: CK, kaolin; CKH, kaolin with heparinase; LMWH, low-molecular-weight heparin; NPV, negative predictive value; PPV, positive predictive value; R, reaction time; UFH, unfractionated heparin.

### The Effect of LMWH on TEG 6s Parameters

**Fig. 2:**
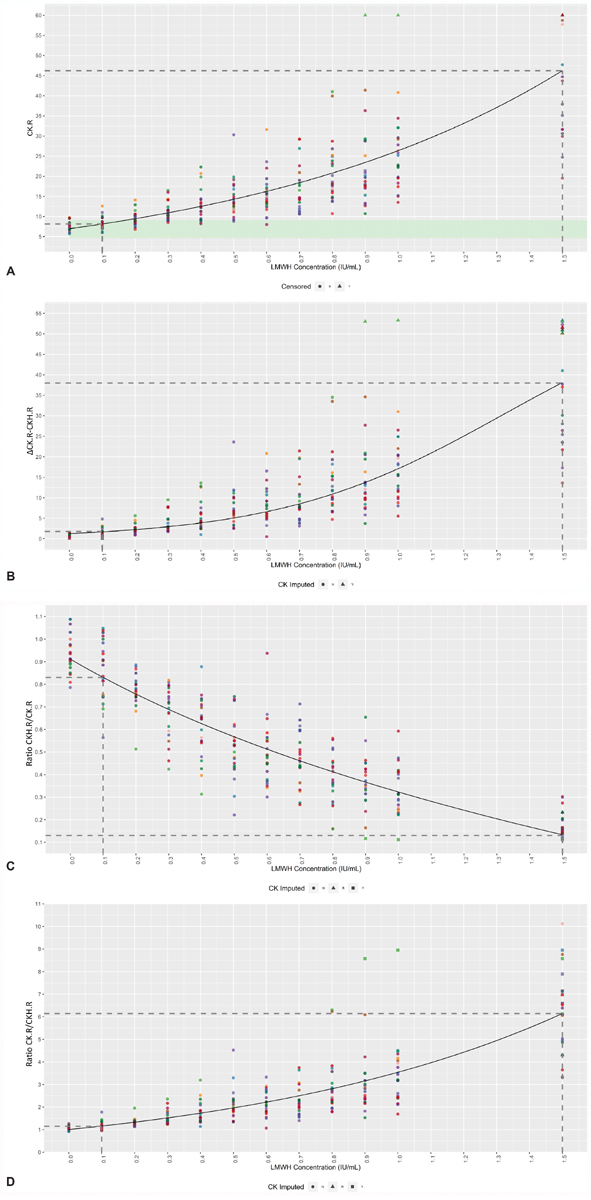
Concentration-dependent effects of LMWH on the TEG 6s parameters. Mixed-effect four-parameter logistic model of ( **A** ) CK.R, ( **B** ) ΔCK.R − CKH.R, ( **C** ) ratio CKH.R/CK.R, and ( **D** ) ratio CK.R/CKH.R in relation to the LMWH concentration. Individual donors are highlighted in different colors, with the model ( *black line* ) included. Reference range limits for CK.R are shown in *light green* . The *dotted lines* represent the minimum and maximum values (first dose where the response was statistically significant from the dose 0 response or last dose where the response was statistically different from the last observed dose in the model, respectively). Values greater than 60 are beyond the limit of detection, and so are marked as censored. CK, kaolin; CKH, kaolin with heparinase; LMWH, low-molecular-weight heparin; R, reaction time.

Similar to UFH, concentration-dependent effects of LMWH were shown for CK.R ( *p* < 0.001), with readings within the reference range limits for therapeutic levels of heparin ( Fig. 2A ). A positive Spearman correlation of *ρ* = 0.861 ( *p* < 0.001) was observed, confirming that higher concentrations of LMWH were associated with higher CK.R results. A significant difference from the reference range was first observed at 0.3 IU/mL. Although a significant effect of LMWH on CKH.R was observed, this was not clinically significant as the change remained within the normal reference range as shown by the very low slope (slope *β* = 0.265, *p* = 0.004). Higher concentrations of LMWH also resulted in higher ΔCK.R − CKH.R and ratio CK.R/CKH.R results, as shown by the positive Spearman correlations of *ρ* = 0.879 ( *p* < 0.001) and *ρ* = 0.905 ( *p* < 0.001), respectively ( Fig. 2B, D ), while increasing concentrations of LMWH resulted in lower results for ratio CKH.R/CK.R, demonstrated by a negative Spearman correlation of *ρ* = −0.905 ( *p* < 0.001; Fig. 2C ).

Accurate measurement of LMWH activity could be conducted in the range of 0.1 to 1.5 IU/mL for ratio CKH.R/CK.R, ratio CK.R/CKH.R, and ΔCK.R − CKH.R ( Table 2 ).

The accuracy at which samples were categorized in the clinically relevant therapeutic range (0.3–0.7 IU/mL) for LMWH was lower than that for UFH, at 80% for ratio CKH.R/CK.R, 78% for ratio CK.R/CKH.R, and 79% for ΔCK.R − CKH.R ( Table 3 ). TEG 6s positively identified samples in the therapeutic window of anti-Xa (0.5–1.2 IU/mL) with a PPV of 71% for ratio CKH.R/CK.R, and 68% for both ΔCK.R − CKH.R and ratio CK.R/CKH.R. Samples not in this range were identified with a NPV of >89% for ratio CKH.R/CK.R, >86% for ratio CK.R/CKH.R, and >87% ΔCK.R − CKH.R. A sensitivity of 68% for ratio CKH.R/CK.R and 65% for both ratio CK.R/CKH.R and ΔCK.R − CKH.R were observed for samples within the therapeutic window, with values of >85% for ratio CKH.R/CK.R, >80% for ratio CK.R/CKH.R, and >82% for ΔCK.R − CKH.R for samples outside this window. Specificity was 86% for ratio CKH.R/CK.R and 85% for both ratio CK.R/CKH.R and ΔCK.R − CKH.R at therapeutic levels of LMWH; for samples outside of the therapeutic range, specificity was >88% for ratio CKH.R/CK.R, 90% for ratio CK.R/CKH.R, and >91% for ΔCK.R − CKH.R.

### Use of CRT.ACT and CFF.MA at High Concentrations of Heparin

**Fig. 3:**
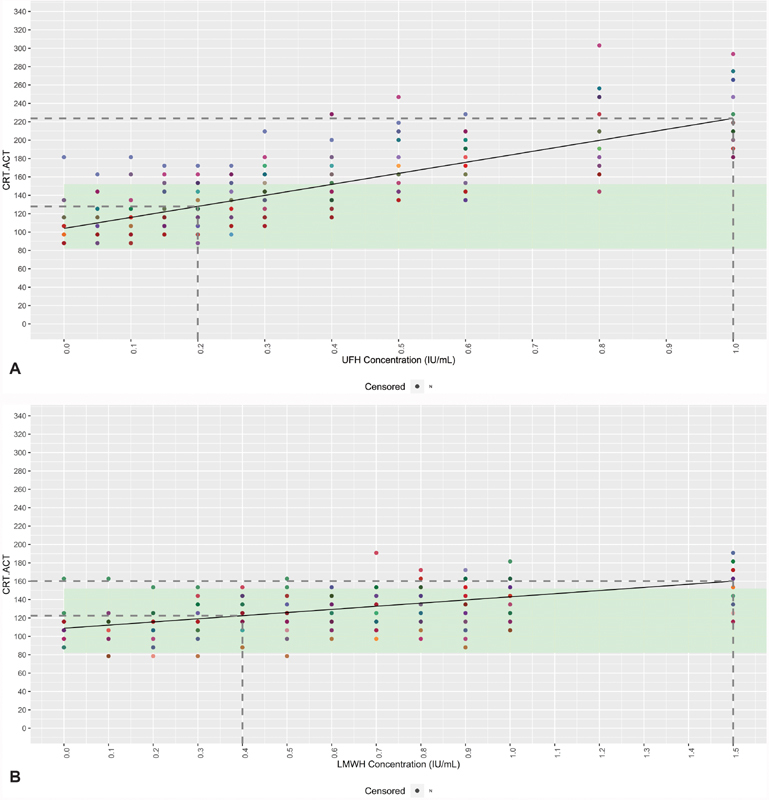
Linear mixed-effects model of CRT.ACT or CFF.MA in relation to the UFH ( **A** ) or LMWH ( **B** ) concentration. Individual donors are highlighted in different colors, with the model ( *black line* ) included. Reference range limits are shown in *light green* . The *dotted lines* represent the minimum and maximum values (first dose where the response was statistically significant from the dose 0 response or last dose where the response was statistically different from the last observed dose in the model, respectively). Values greater than 60 are beyond the limit of detection, and so are marked as censored. ACT, activated clotting time; CFF, citrated functional fibrinogen; CRT, RapidTEG; LMWH, low-molecular-weight heparin; MA, maximum amplitude; UFH, unfractionated heparin.

Significant concentration-dependent effects of both UFH and LMWH were also shown on CRT.ACT results (UFH, slope *β* = 119.750, *p* < 0.001; LMWH, slope *β* = 34.170, *p* < 0.001; Fig. 3 ). Higher concentrations of both UFH and LMWH correlated with higher results for CRT.ACT, with positive Spearman correlations of *ρ* = 0.798 and *ρ* = 0.560 ( *p* < 0.001 for both), respectively. The concentration at which a significant difference to the reference range was first observed was 0.5 IU/mL for UFH and 1.5 IU/mL for LMWH.

CFF.MA also showed a statistically significant correlation to UFH ( Supplementary Fig. 1A ) and LMWH ( Supplementary Fig. 1B ) concentration (UFH, slope *β* = − 3.16, *p* < 0.001; LMWH, slope *β* = − 1.31, *p* < 0.001). In both cases, the correlation was negative, with higher concentrations of UFH or LMWH resulting in a lower CFF.MA and Spearman correlations of *ρ* = − 0.207 and *ρ* = − 0.148, respectively ( *p* < 0.001 for both). However, the slope was minimal, and the mean change remained within the assay reference range up to 1 IU/mL for UFH and 1.5 IU/mL for LMWH. At higher concentrations of heparin, CFF.MA did not follow a linear trend and coagulation became impaired, with a notable lowering of CFF.MA. The minimum value at which heparin could be accurately quantified was 0.8 IU/mL for UFH and 1.0 IU/mL for LMWH.

## Discussion

This study suggests that the TEG 6s system can be used effectively to monitor anticoagulation and hemostasis within therapeutic ranges for both UFH and LMWH, and can accurately classify 78 to 92% of blood samples according to clinically relevant levels of heparin. TEG 6s generates initial results within minutes and full results within 30 to 60 minutes 35 36 37 from a single multichannel cartridge, while providing an accurate method of monitoring the level of anticoagulation with both UFH and LMWHs. This is in contrast to standard laboratory tests that take 45 to 60 minutes. 38

Concentration-dependent effects of increasing heparin on TEG 6s parameters have been observed, with the exception of those assays containing heparinase. 24 39 40 41 From our results, a higher concentration of heparin tended to be associated with a larger CK.R value, and was significantly associated with an increase in CRT.ACT readings. In agreement with these results, publications using previous TEG models have also reported that the R is prolonged by increasing UFH and LMWH doses. 18 25 39 The R was prolonged in >90% ( *n* = 47/50) coronary care unit patients receiving enoxaparin, and correlated with the dose per kg. 25 Additionally, a strong correlation between R parameters and anti-Xa levels was reported in a study of seven healthy volunteers injected subcutaneously with dalteparin, leading the study authors to conclude that R was a suitable basic parameter for clinically monitoring LMWH. 27 In the ECMO setting, a retrospective study of 31 patients receiving UFH recommended combining R and ACT results to guide changes in heparin dose, 25 and a prospective study on 42 patients demonstrated that R can be safely used to guide anticoagulation management. 29

Here we also show that TEG 6s R-time parameters CK.R and CKH.R can be combined to classify samples according to therapeutic ranges of UFH and LMWH, as determined by anti-Xa assay results. Calculating either the difference between CK.R and CKH.R parameters or a ratio of these parameters (ratio CK.R/CKH.R; ratio CKH.R/CK.R) increased the sensitivity to UFH and LMWH, and these combined parameters could be more sensitive to very low concentrations of UFH than the anti-Xa assay. 19 Our results show that ΔCK.R − CKH.R, ratio CK.R/CKH.R, and ratio CKH.R/CK.R metrics can all accurately categorize samples into therapeutic ranges for both UFH and LMWH. The accuracy at which samples were categorized with ΔCK.R − CKH.R was highest at 92%, closely followed by ratio CK.R/CKH.R at 90% and ratio CKH.R/CK.R at 91%. In the case of LMWH, the accuracy was lower at >78% overall, with the highest accuracy observed for ratio CKH.R/CK.R at 80%. There are few published studies that evaluate composite TEG parameters, although those available have also demonstrated an excellent correlation with anti-Xa levels when compared with the TEG parameters alone. 27 28

The quantification window within which heparin could be accurately measured using these combination parameters ranged from the concentration of 0.05 to 0.8 IU/mL for UFH and 0.1 to 1.5 IU/mL for LMWH. For higher levels of heparin, CRT.ACT proved to be a suitable parameter, with a difference from the reference range observed at >0.5 IU/mL for UFH and >1.5 IU/mL for LMWH. Although CFF.MA showed significant negative correlation with both UFH and LMWH, the change remained within the assay reference range up to 1 IU/mL for UFH and 1.5 IU/mL for LMWH, and did not follow a linear trend at higher concentrations of heparin.

It has previously been reported that the TEG 5000 can be used to monitor the antithrombotic effects of UFH and LMWH with greater sensitivity than conventional coagulation tests. 18 19 Results published by Coppell et al indicate that the TEG 5000 may have lower sensitivity when measuring CK.R than the TEG 6s, with the first dose at which a significant difference from the reference range could be observed measured as 0.25 IU/mL for UFH and 0.5 IU/mL for LMWH. 19 In contrast, we demonstrated a difference from the reference range for CK.R at 0.1 IU/mL for UFH and 0.3 IU/mL for LMWH. This difference in sensitivity may be potentially explained by the improved repeatability of results with the TEG 6s due to the increased automatization of sample processing. Coppell et al also investigated the utility of the combination parameter ΔCK.R − CKH.R, noting that this greatly increased the sensitivity of the assay; with this combination parameter the TEG 5000 was able to detect lower doses of UFH and LMWH than those investigated in our study. 19 It would be expected that as the TEG 6s shows greater sensitivity to the effect of heparins on the CK.R assay than the TEG 5000, it would be possible to detect even lower heparin concentrations than those investigated by Coppell et al, but this requires further investigation.

There are a few limitations related to our study design. Due to the addition of heparin, coagulation was delayed or impaired, and the instrument only monitored the coagulation process for 60 minutes. Consequently, parameter results were not produced for some of the assays. Results for those samples were imputed as described in the methods and were marked as “censored” in the statistical analyses and plots. All samples evaluated in this study were obtained from healthy donors and ex vivo dosing, rather than in vivo dosing with samples from heparinized patients. This allowed our study to evaluate the effects of heparin on TEG 6s parameters under well-controlled conditions. The use of spiked healthy blood ensured that we were able to obtain various heparin doses with identical blood samples; when using samples from heparinized patients, it is not possible to control the levels of heparin to the same extent. As whole blood samples were used for the TEG 6s testing, it is possible that additional factors such as platelet count or fibrinogen concentration may affect results; as aPTT and anti-Xa assays are conducted in plasma, this effect would be minimized. It would also not be ethically feasible to subject a healthy donor to escalating doses of heparin to generate suitable blood samples. As this study only included samples from healthy patients, further testing would be advised to validate these results in clinical samples.

In conclusion, this study provides novel information demonstrating that TEG 6s assays can effectively be used to monitor and quantify anticoagulation in situations where heparin is present, even at high therapeutic levels. The use of combination parameters allows for classification of blood samples into therapeutic ranges based on heparin activity, indicating the potential for high clinical utility of the TEG 6s in heparinized patients.
