# Supplementary material for: Cartridge-Based Thromboelastography Can Be Used to Monitor and Quantify the Activity of Unfractionated and Low-Molecular-Weight Heparins
Source: TH Open. 2019 Sep 12;3(3):e295–305. doi: 10.1055/s-0039-1696658 (PMC6742498; doi:10.1055/s-0039-1696658)
Supplement: Supplementary file 1 — Supplementary Material [file 10-1055-s-0039-1696658-s190023.pdf]

Supplementary Table S1 Comparison of the results from the anti-Xa, aPTT, and TEG 6s assays (values are reported as mean [IQR]; shading indicates the therapeutic range)

Spiked concentration of heparin (units)	Anti-Xa	aPTT	CK.R	CKH.R	CRT.ACT	CFF.MA
UFH						
0	0.0 [0.0, 0.0]	32.0 [31.2, 33.1]	7.7 [6.8, 8.2]	6.8 [6.7, 7.4]	97.3 [90.3, 116.0]	19.3 [17.6, 21.1]
0.05		33.9 [33.0, 35.2]	9.6 [8.2, 10.5]	7.8 [7.2, 8.4]	106.6 [97.3, 116.0]	19.1 [18.0, 20.9]
0.1		39.0 [37.8, 42.3]	11.6 [10.3, 14.3]	7.6 [7.4, 7.9]	106.6 [97.3, 116.0]	19.2 [17.9, 21.2]
0.15	0.2 [0.2, 0.2]	48.3 [44.5, 52.1]	15.9 [13.4, 20.0]	7.4 [7.0, 7.8]	106.6 [106.6, 116.0]	19.3 [17.3, 20.8]
0.2	0.3 [0.2, 0.3]	58.1 [51.1, 62.4]	19.2 [15.7, 24.0]	7.1 [6.4, 8.0]	120.7 [106.6, 132.3]	18.9 [17.0, 20.9]
0.25	0.4 [0.3, 0.4]	70.4 [59.0, 74.6]	24.8 [19.5, 30.5]	7.2 [6.6, 7.7]	120.7 [106.6, 125.3]	19.1 [16.6, 21.1]
0.3	0.4 [0.4, 0.5]	83.8 [66.2, 88.0]	32.7 [25.0, 37.7]	7.6 [6.7, 8.0]	134.7 [118.3, 158.0]	18.9 [16.3, 20.9]
0.4	0.7 [0.5, 0.7]	114.2 [86.6, 120.2]	46.8 [35.8, 49.5]	7.5 [6.6, 7.8]	139.3 [134.7, 158.0]	19.2 [15.6, 20.8]
0.5	0.9 [0.8, 0.9]	143.9 [113.2, 153.4]	47.0 [43.3, 52.0]	7.2 [6.8, 7.6]	162.7 [155.7, 179.2]	18.2 [16.9, 20.0]
0.6	1.1 [0.9, 1.1]	169.5 [130.7, 173.0]	48.5 [44.4, 58.6]	7.2 [7.0, 7.8]	162.7 [144.0, 179.2]	18.3 [15.8, 20.2]
0.8	1.4 [1.3, 1.5]	171.6 [170.1, 172.7]		7.3 [7.0, 7.9]	204.8 [165.0, 242.2]	17.6 [14.7, 19.6]
1	1.7 [1.6, 1.8]			8.0 [7.0, 8.4]	209.5 [202.5, 237.6]	17.4 [14.6, 19.1]
2				7.8 [7.2, 8.2]	340.5 [303.1, 373.2]	13.5 [9.4, 16.9]
3				7.8 [7.3, 8.1]	466.8 [415.3, 527.6]	10.1 [4.1, 14.2]
4				8.2 [7.5, 8.5]	593.0 [464.5, 1367.2]	8.7 [3.3, 14.6]
5				7.8 [7.5, 8.2]	733.4 [651.6, 1187.1]	2.9 [2.8, 4.7]
6				8.3 [8.0, 8.6]	1135.7 [1014.0, 1233.9]	2.8 [2.8, 2.8]
LMWH						
0	0.0 [0.0, 0.0]	30.6 [28.7, 32.6]	7.5 [6.6, 8.1]	7.2 [6.5, 7.8]	106.6 [97.3, 113.7]	19.8 [18.0, 21.5]
0.1		33.9 [31.1, 35.8]	7.9 [7.0, 8.7]	7.2 [6.7, 7.8]	111.3 [97.3, 116.0]	19.5 [17.7, 21.9]
0.2	0.4 [0.4, 0.4]	37.3 [34.0, 38.9]	9.6 [8.5, 10.5]	7.1 [6.7, 7.8]	106.6 [99.6, 113.7]	19.9 [17.9, 22.3]
0.3	0.6 [0.5, 0.6]	42.0 [37.4, 43.8]	10.1 [9.4, 11.3]	7.6 [6.2, 8.6]	116.0 [108.9, 134.7]	19.5 [18.0, 21.9]
0.4	0.8 [0.7, 0.8]	45.4 [40.6, 47.6]	11.8 [10.7, 14.0]	7.5 [7.3, 7.9]	120.7 [116.0, 139.3]	19.3 [17.7, 21.6]
0.5	0.9 [0.9, 1.0]	48.8 [43.4, 52.2]	12.9 [11.4, 14.8]	7.3 [7.0, 8.0]	116.0 [108.9, 123.0]	19.7 [17.6, 21.7]
0.6	1.1 [1.1, 1.2]	52.1 [46.6, 56.4]	15.6 [12.9, 17.1]	7.3 [6.6, 7.6]	116.0 [108.9, 141.7]	19.4 [17.6, 21.6]

Supplementary Table S1 (*Continued*)

Spiked concentration of heparin (units)	Anti-Xa	aPTT	CK.R	CKH.R	CRT.ACT	CFF.MA
0.7	1.3 [1.2, 1.4]	56.0 [50.2, 61.8]	16.2 [14.4, 20.4]	7.4 [6.9, 8.0]	116.0 [108.9, 137.0]	19.6 [17.6, 21.4]
0.8	1.5 [1.4, 1.5]	60.0 [54.4, 66.8]	18.1 [16.7, 24.8]	7.6 [6.5, 8.0]	125.3 [116.0, 132.3]	19.2 [17.2, 20.6]
0.9	1.6 [1.5, 1.7]	63.1 [56.5, 69.3]	19.2 [15.7, 27.8]	7.1 [6.4, 8.1]	130.0 [108.9, 141.7]	19.4 [17.1, 20.4]
1	1.8 [1.6, 1.8]	67.9 [59.6, 74.6]	25.1 [19.3, 29.5]	7.7 [7.1, 9.1]	125.3 [116.0, 151.1]	18.5 [17.2, 20.6]
1.5	1.9 [1.9, 1.9]	94.7 [86.4, 108.7]	38.2 [30.4, 50.5]	7.0 [6.7, 8.9]	158.1 [139.4, 169.8]	18.8 [16.6, 19.9]
2		123.3 [116.0, 145.1]	55.0 [54.6, 55.5]	8.8 [7.8, 9.5]	167.4 [148.7, 181.5]	17.8 [15.6, 19.3]
2.5		155.7 [144.5, 163.9]	22.3 [22.3, 22.3]	8.6 [7.9, 9.3]	172.1 [165.0, 179.2]	17.6 [15.6, 18.3]
5			55.3 [55.3, 55.3]	8.6 [8.3, 10.8]	218.8 [202.5, 263.3]	16.4 [15.6, 17.1]
10				9.8 [9.1, 11.2]	232.9 [188.5, 254.0]	15.2 [14.3, 16.2]

Abbreviations: ACT, activated clotting time; aPTT, activated partial thromboplastin time; CFF, functional fibrinogen; CK, kaolin; CKH, kaolin with heparinase; CRT, RapidTEG; LMWH, low-molecular-weight heparin; MA, maximum amplitude; R, reaction time; UFH, unfractionated heparin.

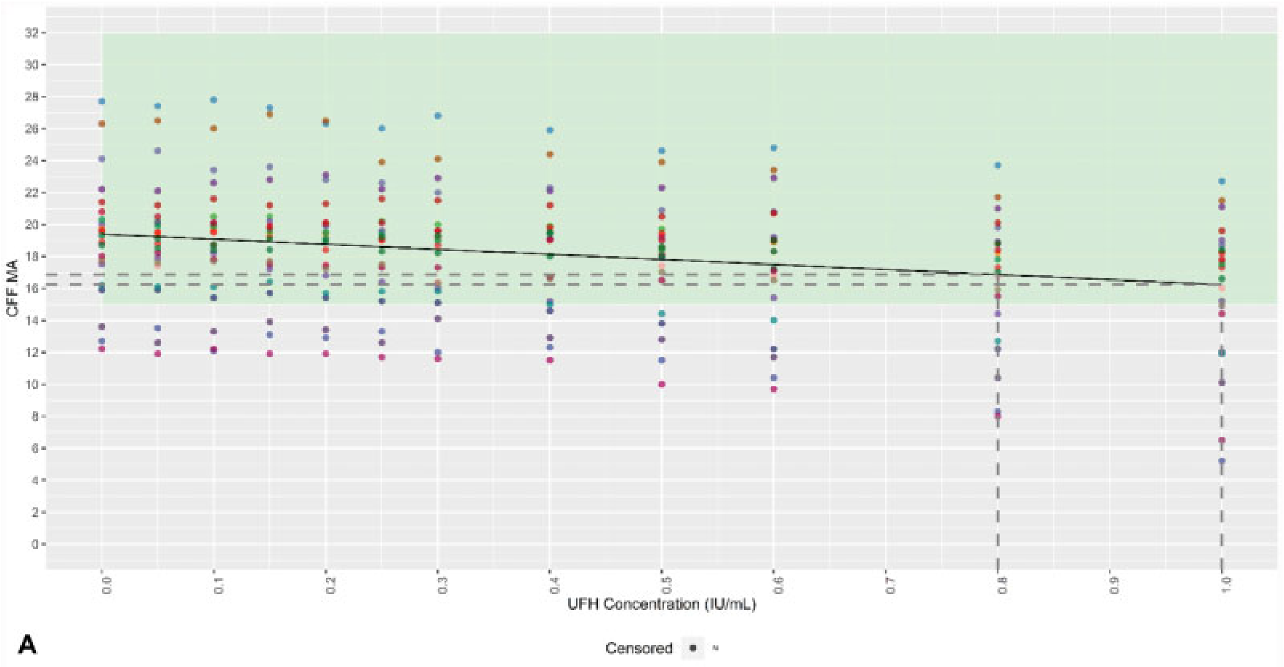

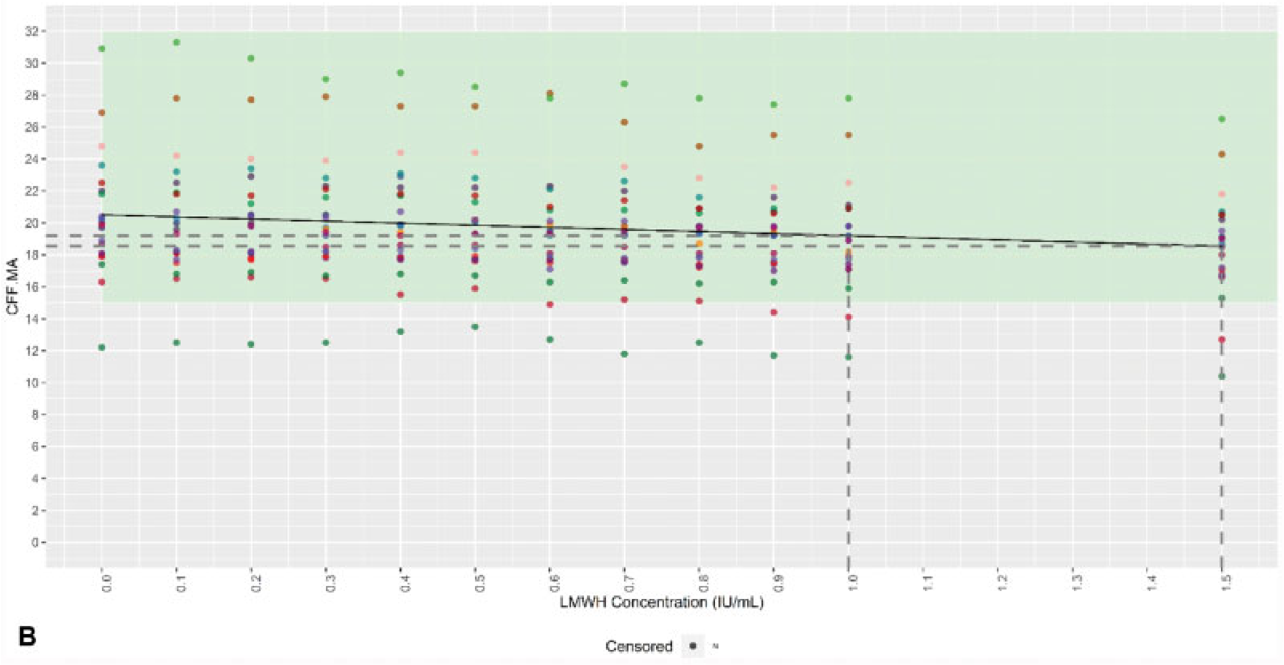

Supplementary Fig. S1 Linear mixed-effects model of CFF.MA in relation to the UFH (**A**) or LMWH (**B**) concentration. Individual donors are highlighted in different colors, with the model (*black line*) included. Reference range limits are shown in *light green*. The *dotted lines* represent the minimum and maximum values (first dose where the response was statistically significant from the dose 0 response or last dose where the response was statistically different from the last observed dose in the model, respectively). Values greater than 60 are beyond the limit of detection, and so are marked as censored. CFF, citrated functional fibrinogen; LMWH, low-molecular-weight heparin; MA, maximum amplitude; UFH, unfractionated heparin.
